# Molecular Dynamics Simulations in Designing DARPins as Phosphorylation-Specific Protein Binders of ERK2

**DOI:** 10.3390/molecules26154540

**Published:** 2021-07-27

**Authors:** Vertika Gautam, Piyarat Nimmanpipug, Sharifuddin Md Zain, Noorsaadah Abd Rahman, Vannajan Sanghiran Lee

**Affiliations:** 1 Department of Chemistry, Faculty of Science, University of Malaya, Kuala Lumpur 50603, Malaysia; vartikapisces@gmail.com (V.G.); smzain@um.edu.my (S.M.Z.); noorsaadah@um.edu.my (N.A.R.); 2 Department of Chemistry, Faculty of Science, Chiang Mai University, Chiang Mai 50200, Thailand; piyarat.n@cmu.ac.th; 3 Center of Excellence for Innovation in Analytical Science and Technology (I-ANALY-S-T), Chiang Mai University, Chiang Mai 50200, Thailand

**Keywords:** molecular dynamics simulations, extracellular regulated kinase, DARPins, MAESTRO

## Abstract

Extracellular signal-regulated kinases 1 and 2 (ERK1/2) play key roles in promoting cell survival and proliferation through the phosphorylation of various substrates. Remarkable antitumour activity is found in many inhibitors that act upstream of the ERK pathway. However, drug-resistant tumour cells invariably emerge after their use due to the reactivation of ERK1/2 signalling. ERK1/2 inhibitors have shown clinical efficacy as a therapeutic strategy for the treatment of tumours with mitogen-activated protein kinase (MAPK) upstream target mutations. These inhibitors may be used as a possible strategy to overcome acquired resistance to MAPK inhibitors. Here, we report a class of repeat proteins—designed ankyrin repeat protein (DARPin) macromolecules targeting ERK2 as inhibitors. The structural basis of ERK2–DARPin interactions based on molecular dynamics (MD) simulations was studied. The information was then used to predict stabilizing mutations employing a web-based algorithm, MAESTRO. To evaluate whether these design strategies were successfully deployed, we performed all-atom, explicit-solvent molecular dynamics (MD) simulations. Two mutations, Ala → Asp and Ser → Leu, were found to perform better than the original sequence (DARPin E40) based on the associated energy and key residues involved in protein-protein interaction. MD simulations and analysis of the data obtained on these mutations supported our predictions.

## 1. Introduction

Protein kinases play a principal regulatory role in nearly all aspects of cell biology. The human genome encodes 538 protein kinases [1]. Out of all the post-translational modifications (PTMs), protein phosphorylation is the most widespread class used in signal transduction. One of the members of the protein kinase family, mitogen-activated protein kinase (MAPK) is a type of protein kinase that is specific to the amino acids serine and threonine (i.e., a serine/threonine-specific protein kinase). MAPK is involved in the most fundamental pathway to cell biology, known as the MAPK pathway, and it plays a crucial role in integrating cell surface signals to transcriptional regulation of the proteome [2]. The MAPK pathway is also referred to as the RAS-RAF-MEK-ERK signal cascade. The main function of this cascade is to regulate physiological processes by transmitting upstream signals to its downstream effectors. They are mainly involved in cell differentiation, proliferation, survival, and death. Targeting the MAPK pathway is thought to be a hopeful strategy for cancer therapy as it is the most often mutated signalling pathway in human cancer. In the past decades, extensive efforts have been made by different research groups, leading to the clinical success of BRAF and MEK inhibitors. 

In the early 1980s, the first protein kinase inhibitor was developed followed by FDA approval of many more drugs as kinase inhibitors for the treatment of cancers, for example, lung and breast cancers. Till date, around 150 drugs targeting kinases have undergone clinical phase trials, and many other specific inhibitors are in the preclinical stage of drug development [3]. However, with the speedy increase in resistance developed to clinical RAF and MEK inhibitors, interest has been encouraged towards targeting ERK directly for cancer therapy.

Natural proteins and antibodies undoubtedly changed medicine, but they usually treat only one disease target. Antibodies have some well-known limitations such as expensive production, difficult formulation, low tissue penetration, and complex architecture, and they bind to their target bivalently. On the other hand, designed ankyrin repeat proteins (DARPins) have completely overcome these limitations of conventional therapeutic approaches due to their small size, high stability, high potency, high affinity (strong binding), and flexible architecture. Moreover, the development of in vitro selection technologies such as ribosome display [4] and phage display [5] has enabled the selection of DARPins as specific binders. DARPins show commendable stability (thermodynamic as well as intracellular), which has made them promising candidates for therapeutic applications [6,7,8,9,10].

**Figure 1:**
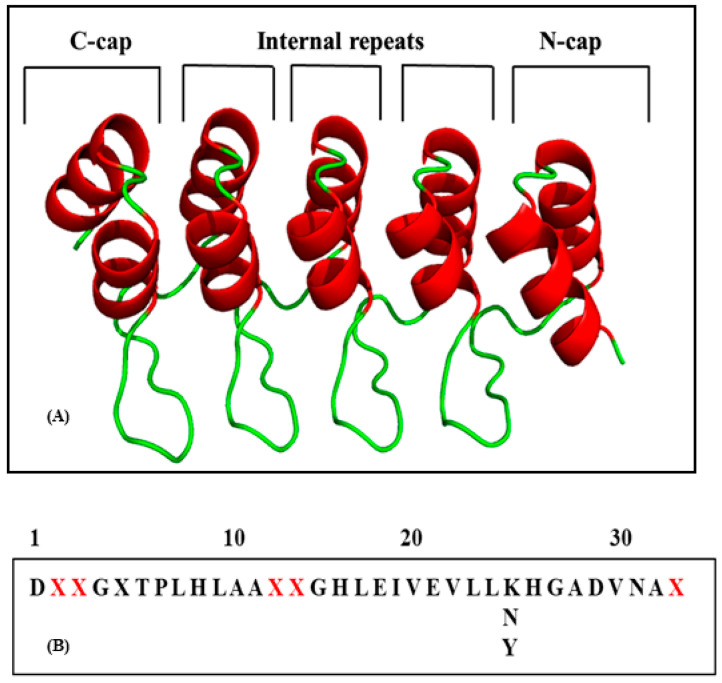
(**A**) Architecture of DARPins (N3C) showing *C*-cap, *N*-cap, and internal repeats. (**B**) Consensus design of a DARPin repeat containing 33 amino acids showing framework (black) and randomized residues (red).

Based on naturally occurring ankyrin repeat proteins, DARPins are emerging a promising new class of binding proteins [11,12]. DARPins (designed ankyrin repeat proteins) are one of the most profusely found binding proteins in the human genome [13]. The structure of ankyrin repeat proteins consists of tightly joined repeats of 33 amino acid residues [14]. The basic structural unit of ankyrin repeats consists of two antiparallel α-helices preceding a β-turn. In a single protein, up to 29 consecutive repeats can be found [15,16]. DARPins repeat a typical structure that consists of a module flanked by *N* and *C* caps (Figure 1A), where *N*- and *C*- designate *N* and *C* terminal capping repeats, respectively, and “–” stands for the number of library modules that ranges between 2 and 4 (N2C and N3C) [17]. The hydrophobic core of “repeats” is shielded by these caps. Domains of ankyrin repeats forming a continuous hydrophobic core together with a large solvent-accessible surface that holds the repeat modules together provide stability to the structure. They are thermodynamically very stable. Through a consensus approach, it was found that repeats of DARPin consist of residues that are responsible for the maintenance of its structure (fold conservation) called fixed framework residues, while there are other residues through which DARPins interact with their target proteins known as randomized interacting residues (Figure 1B) [14]. Variations in DARPins can be brought about through randomized residues along with conserved interfaces that are present between repeat units. These interfaces are the places where single repeats can be deleted, inserted, or exchanged, maintaining the tertiary structure intact [18]. In addition to designing a large DARPin library, the consensus design approach also produced desired DARPins with enhanced properties in terms of expression levels, stability, and solubility [12,17]. All these qualities make them a suitable candidate for ERK inhibition. In the present study, we have explored DARPins (designed ankyrin proteins) as ERK2 inhibitors.

In the pharmaceutical industry, computational drug design has played a vital role in the discovery, design, and analysis of drugs. Computer technology nowadays is so rich and advanced that the accuracy of biomolecular simulations is consistently high enough to be used to truly drive preclinical drug discovery projects. Among the computational tools used for drug discovery, molecular dynamics simulations (MDS) and related methods are routinely used nowadays. Their main contribution is towards the understanding of structural flexibility together with entropic effects of complex systems. This allows an in-depth estimation of the thermodynamics and kinetics related to drug-target recognition and binding [18]. MD simulations have been extensively used in the study of protein-protein and protein-ligand interactions, and the study of the mechanism of drug action [19,20,21,22,23]. The plethora of applications of molecular dynamics simulations extend from the study of complex and dynamic processes that play a central role in biological systems to the structure determination from X-ray crystallography and NMR experiments. In biological systems, the main application of MDS focuses on studies related to the stability of proteins through conformational changes and folding. MD studies also enable the molecular recognition of cellular components such as DNA, membranes along with complexes (drug–receptor), and ion transport [24,25,26,27]. MD studies have also enabled the study of the mechanism of drug resistance [28,29,30,31]. Molecular dynamics simulations calculate the binding free energy, which is very helpful in investigating receptor-ligand interactions [32]. Over time, MDS have made immense contributions towards drug discoveries, exploring ligands such as small molecules, chemicals derived from plants, peptides, and proteins against targets such as protein kinases (PKs) [33,34,35,36,37], G-protein-coupled receptors (GPCRs) [38], and NMDA receptors [20,39,40]. Altogether, this method affords a means for drug design by providing a holistic approach to understanding the mechanism of receptor activation/deactivation, inhibiting the receptor to the mechanism of drug resistance.

During MD simulations, configurations of the progressing system are sequentially generated; these configurations come out as trajectories that contain specific details of the positions and velocities of a particle over the time of simulation. These trajectories are explored to calculate a variety of properties, such as kinetic measures and free energy and other macroscopic quantities. These properties can be further compared with experimental observables that are helpful in drug design. This method was initially perceived within theoretical physics in the late 1950s, and its application was extended to chemical physics, materials science, biomolecular modelling, and, more recently, drug discovery [41].

In the past decade, there has been immense progress in the development of algorithms and technology using mutations, which has transformed the field of protein design and engineering to attain tailormade proteins suitable for pharmaceutical and biotechnological applications. Among various in silico tools available, the Cologne University Protein Stability Analysis Tool (CUPSAT) [42], Site Directed Mutator (SDM) [43], PopMusic 2.1 [44], SNPeffect 4.0 [45], PolyPhen-2 [46], DUET [47], MAESTRO Web [48], DynaMut [49], and mCSM PPI2 [50] for mutational studies have been successfully used to evaluate stability change (stabilizing or destabilizing) and, after mutations, to predict the phenotypic consequence of missense variants. These tools are structure based, sequence based, and energy based, and combined features (statistical approach and/or machine learning methods, such as neural networks and support vector machines (SVM). These methods are fast, user-friendly, and reliable and promise to be invaluable in the development of proteins with a wide range of impactful applications. The applications of these tools extend from understanding the origin of diseases caused by misregulation of protein maintenance [51,52] to discriminating disease-associated mutations from non-disease mutations, studying drug-resistant mutations [53,54,55], and providing important structural and functional insights into designing new proteins [56,57,58,59]. To design DARPins as ERK2 inhibitors, a multi-point mutation approach, MAESTRO, was applied to the wild-type DARPin protein to identify the stabilizing mutation points, followed by validation of the binding energy of mutants employing MD simulations using MM-PBSA/GBSA protocols. The details of the results are discussed in the next section.

## 2. Results

### 2.1. Design and Prediction of New Inhibitors

**Figure 2:**
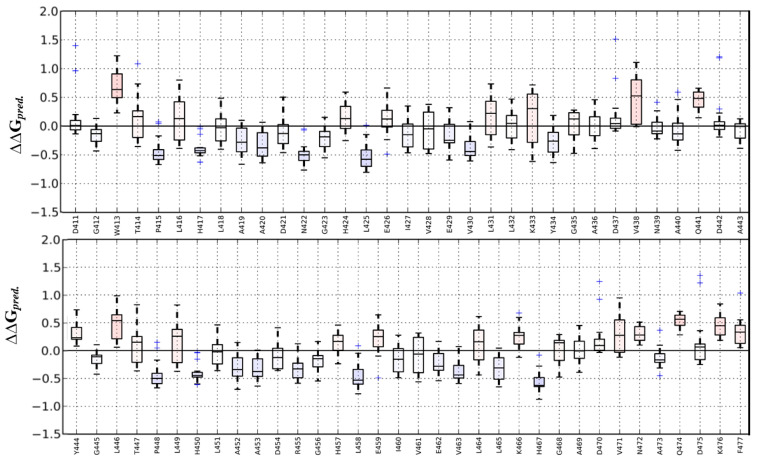
Mutation-sensitive profile of the complex E40/ERK2 obtained from MAESTRO. Confidence estimation and prediction error on multi-point mutations are shown. Blue and red bars show stabilizing and destabilizing mutations, respectively.

The effect of mutations on the thermodynamic stability of DARPins (E40) was analysed using MAESTRO and the predictions were further analysed using other algorithms, and a comparison of ΔΔG predictions is shown in Table S1. MAESTRO is an easy and standalone program that provides different kinds of mutation experiments on single chains and protein complexes. The predictive power of this method is suggested to be reliable as it combines multiple linear regression (MLR), a neural network approach (NN), and a support vector machine (SVM) that allows to include additional information such as protein size or solvent accessibility. The mutation sensitivity profile of E40 is shown in Figure 2. Mutation points were selected based on MAESTRO suggestions, from which only randomized residues of DARPin E40 were chosen to undergo further investigation; refer to Figure 1B. 

#### 2.1.1. Evaluation of Specific Mutations

In total, seven single-point favourable mutations (S380, I389, D421, N422, A443, D454, and R455) were suggested by MAESTRO based on predicted change in stability and confidence estimation calculated in terms of ∆∆G and C*_pred_*, respectively. Wild-type amino acids (AA) in DARPin E40 were mutated with 16 other available amino acids (except cysteine, proline, and glycine). Table 1 shows the suggested mutations with their respective ∆∆G*_pred_* and C*_pred_*. 

#### 2.1.2. Evaluation of Selected Mutants by MDS

In the light of these results, out of all the suggested mutation points from MAESTRO, a total of 13 mutation points meeting the criterion, i.e., ∆∆G < 0.0 (stabilizing) and C*_pred_* values ~1 (highly reliable), were selected for further analysis. Their structures were modelled using the “Build mutant” protocol in DS Modeller [60], further optimized, and finally subjected to large-scale MD simulations to investigate the structural consequences of mutating residues. To study the forces interplay, trajectories of mutant complexes were analysed for binding free energy, per-residue binding free energy of complexes, and pairwise binding free energy of residues within 4 Å.

The trajectories obtained from the production run of 100 ns MD simulations of mutants were analysed for their binding free energy. Binding free energies of mutant complexes (A443D/ERK2, S380L/ERK2, A443N/ERK2, and N422T/ERK2) suggested better binding than E40/ERK2 (Table 2. In the next section, the decomposition of binding energy and important interacting residues with their H-bonds are discussed in detail. MAESTRO gave the highest match with other prediction techniques and has been used as a guide for the new design.

For ranking MM-GBSA, binding energy criteria are used and the energy contributions are G_gas_ and G_solv_. The polar and non-polar contributions are EGB and ESURF, respectively, for the GB calculations shown in Table 3. Although the electrostatic contribution from all mutants is big, it is mostly compensated by a large positive polar contribution (EGB), making the total polar contribution (EEL + EGB) positive and hence it is mostly the van der Waals term that contributes towards the total binding free energy.

**Figure 3:**
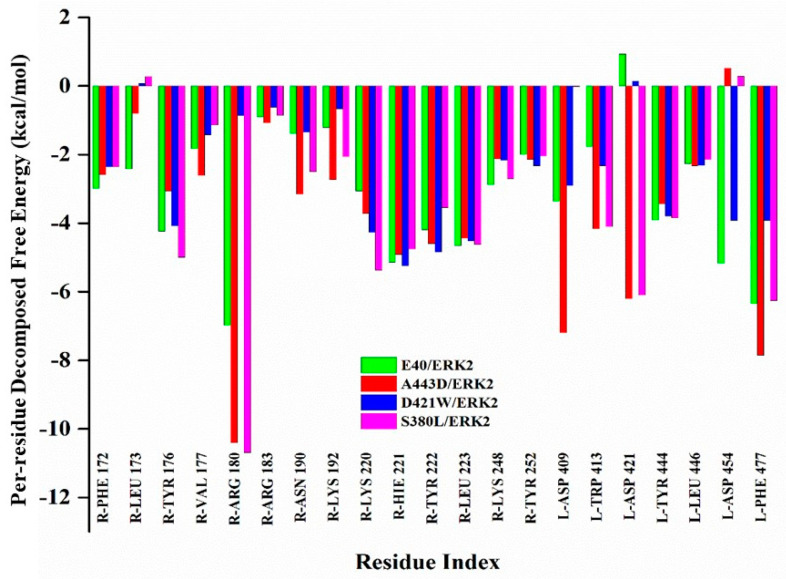
Comparison between the per-residue free energy decomposition of E40/ERK2 and mutants. DARPin residues start with “L”, while ERK2 residues start with “R” followed by a three-letter code of amino acids.

To understand the residue contribution from DARPin and ERK2, a comparison was made for the decomposed free energy for each residue. Important binding residues are shown with their respective energies for all the three mutants, and E40/ERK2.DARPin residues start with “L,” while ERK2 residues start with “R” followed by a three-letter code of amino acids. From Figure 3 it is suggested that most of the important interactions from ERK2 come from the activation loop, specifically ARG180 contributing the most to the αG and L14 regions, while for all the DARPins (N3C), A443D, S380L, and D421W interactions come from all repeats (2,3), including L-ASP409, TRP413, and ASP421, but residues from C-cap (L-TYR444, ASP454, and PHE477) terminal contribute the most. Among all the complexes, A443D/ERK2 and S380L/ERK2 show stronger interactions with decomposed binding free energy <−5 kcal/mol for the above-mentioned residues.

To evaluate the effect of stabilizing mutations on E40 DARPin, the RMSD values of the position differences of backbone atoms between mutant and wild-type structures were calculated throughout the MD simulations. A comparison of the RMSD of E40/ERK2 with the other four complexes pE59/ERK2, A443D/ERK2, S380L/ERK2, and D421W/ERK2 is shown in Figure S1. According to the figures, all the simulations are well converged. According to MAESTRO suggestion, the mutations A443D, S380L, and D421W are stabilizing. After performing 100 ns simulation on the complexes A443D/ERK2, S380L/ERK2, and D421W/ERK2, the average RMSD values are ~3.19, 2.61, and 2.80 Å, respectively.

### 2.2. Exploring the Binding Mechanism of DARPins with ERK2

The main contribution towards the receptor-ligand affinity comes from non-covalent interactions. In the complex A443D/ERK2, alanine at position 443 on the DARPin loop is mutated to aspartate. Alanine contains a non-polar aliphatic R (CH_3_) group, which is small, being mutated to a polar aspartate that has a negatively charged or acidic R (CH_2_COO) group and a long side chain that can enhance electrostatic interactions, which can be seen from the electrostatic energy of −626.46 kcal/mol (Table 3). The second-best selected mutant of E40 that came out after MDS is S380L with a −7 kcal/mol dip in binding free energy (−56.74 kcal/mol) compared to the previous one (Table 2). Serine contains a hydroxymethyl group and is classified as a polar amino acid mutated to a positively charged and non-polar leucine with a side chain containing an isobutyl group. This mutation leads to a more negative electrostatic interaction energy of −673.80 kcal/mol than E40/ERK2 (Table 3). 

To design a high binding inhibitor, it is required to understand interactions that differentiate it from the low binding inhibitors. For this purpose, the third mutant, D421W, having ∆∆G = −41.08 kcal/mol (Table 2), was selected. An aspartic acid at position 421 was mutated to tryptophan that contains a side chain indole, making it non-polar through its aromatic amino acid. Unlike the previous mutations that were located on the DARPin loops, this point mutation on the DARPin repeat has a negative effect on the binding affinity with a binding free energy of −41.08 kcal/mol compared to −49.50 kcal/mol for E40/ERK2.

**Figure 4:**
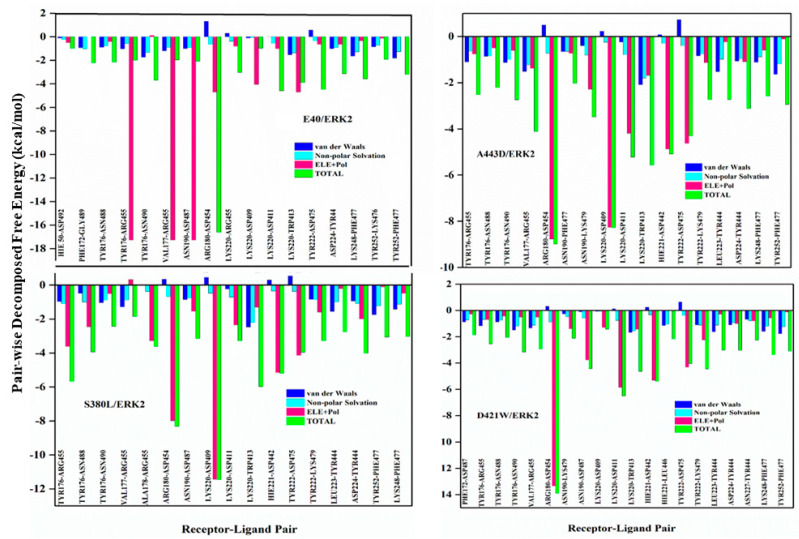
Comparison of pairwise decomposition free energy of complexes. Clockwise: E40/ERK2, A443D/ERK2, D421W/ERK2, and S380L/ERK2. Residues of receptor-ligand pairs and decomposition free energies in kcal/mol are shown on the X and Y axes, respectively. Contributions of van der Waals, non-polar solvation, polar, and total energies are shown with different colour bars.

**Figure 5:**
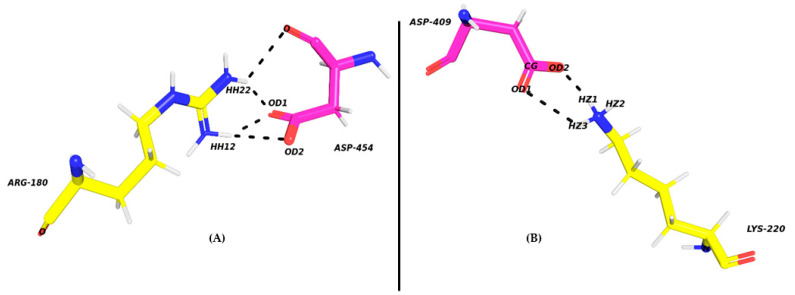
The main salt bridges formed between DARPins and ERK2. (**A**) ARG180-NH2–ASP454-OD1 and (**B**) LYS220 NZ2-ASP409 OD2. Salt bridges are shown by black dots.

**Figure 6:**
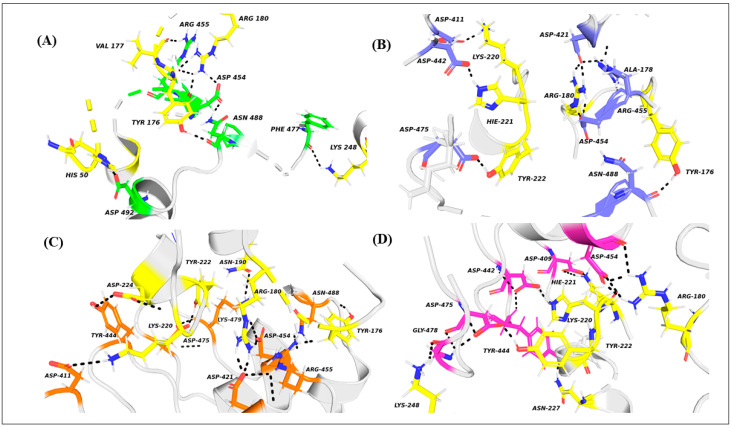
Polar contacts of the complexes of ERK2; residues shown in yellow with (**A**) E40 in green, (**B**) S380L in blue, (**C**) A443D in orange, and (**D**) D421W in pink. Polar interactions are shown by black dotted lines.

Figure 4 displays pairwise decomposition free energy for residues within 4 Å. The DARPin interacts with the receptor mainly through the activation loop, alpha G and MAPK insertion regions [61]. The receptor-ligand interactions are mainly through hydrogen bonds (details of the type of hydrogen bonds are presented in Table 4). For all the three mutants, the hotspot residues show interactions mostly through cation-Π and salt bridges. The two most remarkable salt bridge interactions occur between ARG180-ASP454 and LYS220-ASP409 (Figure 5). In the mutant A443D, salt bridges ARG180-HH12–ASP454-OD1 and LYS220-HZ1–ASP409-OD2 possess quite a low polar interaction energy—(−8.76 and −8.25 kcal/mol) and (−8.98 and −8.27 kcal/mol), respectively; hence the total binding free energy of these residue pairs. There is a remarkable decrease in the energy of LYS220-HZ1–ASP409-OD2 here compared to E40/ERK2. For the next mutant complex S380L/ERK2, the pairwise interaction of 4 Å residues is almost like that in the previous case A443D/ERK2. Here, the interactions between receptor and ligand are also through hydrogen bonds (Table 5) but differ in their energies, with two strong salt bridge interactions between ARG180-HH22–ASP454-OD1 (−8.30 kcal/mol) and LYS220 HZ2-ASP409 OD2. A noticeable drop is observed in the decomposition energy of the receptor-ligand pair LYS220–ASP409 (−11.45 kcal/mol). For the third mutant complex, D421W/ERK2 also follows a pattern similar to that of the pairwise interaction of 4 Å residues like that in the previous cases of A443D and S380L. As in the previous cases, the interactions occur between receptor and ligand through hydrogen bonds, but are not as strong as the previous mutations. Except for a strong salt bridge interaction between ARG180-HH22–ASP454-OD1 (−8.30 kcal/mol), all other interactions have lower ∆∆G values compared to the other two mutants, while salt bridge LYS220 HZ2-ASP409 OD2 shows a remarkable increase (positive) in ∆∆G values. The main polar receptor-ligand interactions of the mutant complexes are shown in Figure 6.

Electrostatic interactions are a pivotal player in protein interactions and helpful in understanding intermolecular protein-protein interactions as they are long-range and have influence on charge molecules. Electrostatic potential maps are also called electrostatic potential energy maps or molecular electrical potential surfaces. These maps demonstrate the charge distributions of molecules in three dimensions and allow us to visualize the variably charged regions of a molecule. Knowledge about the charge distributions can be useful in determining how molecules interact with one another. Moreover, electrostatic forces help in fast recognizing the right partner among hundreds of thousands of candidates present in the intracellular environment of protein-protein complexes [62].

The electrostatic potential surfaces of the complexes E40/ERK2, A443D/ERK2, S80L/ERK2, and D421W/ERK2 were generated using the Adaptive Poisson-Boltzmann Solver (APBS) plugin in PyMOL [63] keeping the same orientation.

To study in detail the specific electrostatic potential of the interacting residues, the interface region in the map was zoomed and the potential was matched with the van der Waals and electrostatic energies of the interacting residues in receptor-ligand pairs.

**Figure 7:**
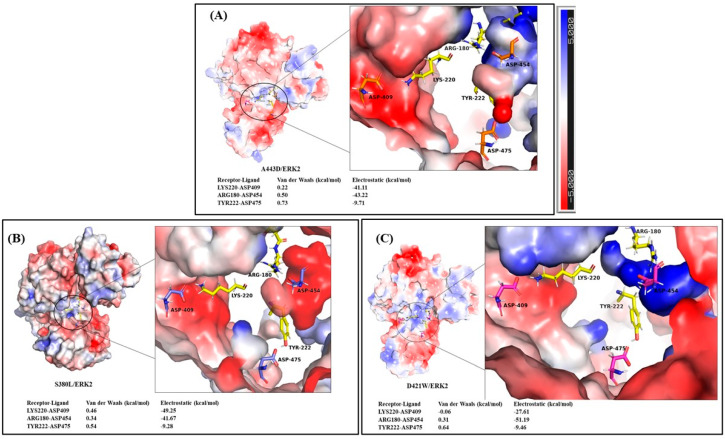
Electrostatic potential surface of interacting residues (enlarged) in (**A**) A443D/ERK2 (**B**) S380L/ERK2, and (**C**) D421W/ERK2. Highly electronegative regions shown by red while electropositive regions are shown by blue color. Respective van der Waals and electrostatic energies for the receptor-ligand pairs are shown in bottom left corner. Red indicates highly electronegative, while blue shows electropositive residue surfaces (see bar). The areas of high electrostatic potential are found near the interface of DARPin and ERK2 in all the structures.

In Figure 7, it can be observed that the interacting residues from receptors and ligands have opposite potentials, i.e., electropositive (blue) and electronegative (red). The electrostatic and van der Waals energy shown in the bottom left corner also corroborates the same. We know that van der Waals are weak forces or temporary attractions between electron-rich regions of one molecule and electron-poor regions of another. For the arginine residue from ARG180, a guanidino group is protonated to give the guanidinium form (-C-(NH_2_)_2_^+^), making arginine a charged, aliphatic amino acid; the lysine from LYS220 is also protonated −NH_3_^+^ at physiological pH, and the tyrosine from TYR222 contains 4-hydroxyphenylalanine that is neutral. These residue charges extend from positive to neutral, which is shown by blue to white regions in the maps. In order to interact, they need to recognize oppositely (negatively) charged residues (aspartate form, −COO^−^) in their vicinity that are ASP409, ASP454, and ASP475, shown by red regions in the maps. ARG180-ASP454 shows the strongest electrostatic energy because of the salt bridge formed between these residues. Although there exist various interactions between receptors and ligands, only important residues are shown in Figure 6. 

In general, single-point mutation in ankyrin does not affect the secondary structure. We have predicted the protein secondary structure for the wild-type and the mutated ankyrin using PredictProtein (ROSTLAB, Technische Universität München, https://predictprotein.org/) [64] and SCRATCH protein predictor (Institute for Genomics and Bioinformatics, University of California, Irvine, USA, http://scratch.proteomics.ics.uci.edu/) [65]. Results are shown in Supplementary Material, Figure S2. It was observed that the secondary structures of mutant DARPins were unaffected.

## 3. Discussion

To design DARPins with higher binding affinity towards ERK2, the MAESTRO method was used to predict stability upon point mutations in N3C DARPin (E40). From the suggested mutation points, 13 stabilizing mutants were subjected to 100 ns simulations and then compared with the wild-type complex (E40/ERK2) in terms of binding free energy. After evaluation, two DARPins, A443D and S380L (showing higher binding affinity than E40), and D421W (showing lower binding than E40) were selected to further analyse their binding mechanism. Out of the suggested mutants, the key elements of the ankyrin mutants with high and low affinity towards ERK2 were compared in terms of binding free energy, decomposition free energy, and strength and type of hydrogen bonds formed between receptor and ligands. It was found that all the DARPins show interactions, mostly coming from their third repeat and *C*-cap terminal residues. The most persistent interactions in each structure were studied via a H-bond analysis, wherein it was found that the high binding affinity of all the N3C DARPins is mainly attributed to salt bridges (ARG180- ASP454 and LYS220-ASP409) along with various other DARPin–ERK2 interactions. Analysis of the energy decomposition shows that electrostatic and van der Waals interactions were the main contributors towards the total binding free energy of these systems. The binding free energy of the newly designed DARPins was improved up to ~20%. The binding affinity of these systems shows a trend of E40 < D421W < S380L < A443D. 

The wild-type DARPin (E40) along with mutants interact (A443D, S380L, and D421W) through hydrogen bonds, mainly salt bridges and cation-Π. Moreover, the strength of the salt bridges formed between ARG180- ASP454 and LYS220-ASP409 plays a significant role in deciding the binding affinity of DARPins towards ERK2. For a DARPin to have a good binding affinity, both interactions must be strong, which can be shown by the order of decomposition free energy of these receptor-ligand pairs and the total binding free energy of the complex. Electrostatic potential surface analysis revealed that mutants that can generate an electronegative potential near the binding interface, show good binding with ERK2. It was also observed that mutations on the loops of DARPins extend better binding compared to that on a DARPin repeat. All this information can be used in the design of new DARPin inhibitors against ERK2.

## 4. Materials and Methods

**Figure 8:**
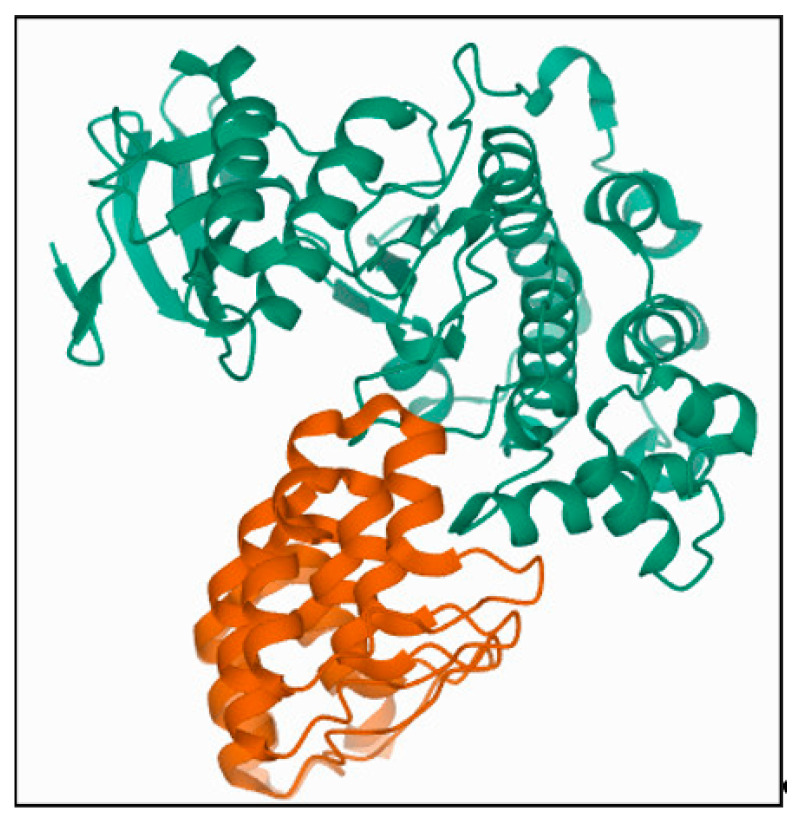
Biological assembly of E40/ERK2 (PDB ID:3ZU7). Ribbon diagrams of DARPin and ERK2 are shown in orange and green, respectively.

The starting structure for the present study is the X-ray crystal structure of DARPin E40 complexed with ERK2 (PDB ID: 3ZU7) [66], designated as E40/ERK2 for further reference in this study. For the complex (E40/ERK2), chain A of ERK2 and chain C of DARPin (E40) were taken as the initial structure for the MD simulations (Figure 8).

MD simulations were performed using the GPU version of Particle Mesh Ewald Molecular Dynamics (PMEMD.CUDA) from AMBER14 [67]. At the molecular level, physical forces were implemented using ff14SB [68] protein force field to carry out MD simulations. The structures were solvated (using the tLeap module implemented in AMBER). The water model TIP3P [69] was used, wherein a cubic box of water extends at least 10 Å from the solute in each direction. A cut-off distance of 15 Å was used to compute the non-bonded interactions (electrostatic interactions and van der Waals interactions). For each of the complexes, a 100 ns long simulation was performed. To minimize the edge effects, all simulations were performed under periodic boundary conditions, and to treat long-range electrostatics, the particle mesh Ewald method was used [70]. To relax the system prior to MD simulation, the complexes were minimized using a series of steepest descent (SD) and conjugated gradient (CG) under the sander module of the AMBER14 program. During the simulation, the system was heated gradually over a period of 60 ps from 0 to 310 K (biological temperature), and a force constant of 5 kcal/mol Å^2^ was applied to restrain the atomic position. A constant pressure of 1 atm for 200 ps (NPT) was applied under Langevin dynamics, followed by a 40 ps volume-constant period (NVT) at a force constant of 2.5 kcal/mol Å^2^, which was maintained and followed by 100 ps dynamics at a force constant of 1.25 kcal/mol Å^2^. Finally, unrestrained production runs were performed for 100 ns, wherein no force was applied on any protein atoms in the NVT ensemble at a constant temperature of 310 K (biological temperature). For all analyses, 500 snapshots were taken from the last 5 ns of the simulation (96–100 ns). To check the system equilibrium, root-mean-square deviation (RMSD) of all backbone atoms was performed using the cpptraj module [71] incorporated in AmberTools 15 and compared to the starting structure. All simulations were carried out under periodic boundary conditions [72]. To treat long-range electrostatics, the particle mesh Ewald method was used [73,74,75], and the SHAKE algorithm was employed to constrain bond lengths involving hydrogen atoms. A 2 fs time step was set up while the trajectory was recorded every 0.1 ps. To relax the system prior to MD simulation, a series of steepest descent (SD) along with conjugated gradient (CG) minimizations with a total of 500 steps each was performed. Post-processing of the trajectories was performed using the MM-PBSA/GBSA protocol [76].

## Figures and Tables

**Table 1:** Specific mutation evaluation using the MAESTRO algorithm.

Mutants	ΔΔG*_pred_*	C*_pred_*	Mutants	ΔΔG*_pred_*	C*_pred_*	Mutants	ΔΔG*_pred_*	C*_pred_*	Mutants	ΔΔG*_pred_*	C*_pred_*	Mutants	ΔΔG*_pred_*	C*_pred_*	Mutants	ΔΔG*_pred_*	C*_pred_*	Mutants	ΔΔG*_pred_*	C*_pred_*
**S380(A)**	0.038	0.959	**N422(A)**	−0.517	0.907	**A443(R)**	0.067	0.807	**D454(A)**	−0.035	0.933	**R455(A)**	−0.146	0.922	**D421(A)**	−0.031	0.926	**I389(Y)**	−0.709	0.861
**S380(R)**	0.402	0.76	**N422(R)**	−0.05	0.851	**A443(N)**	−0.232	0.92	**D454(R)**	0.173	0.801	**R455(N)**	−0.27	0.915	**D421(A)**	0.145	0.808	**I389(W)**	-0.672	0.848
**S380(N)**	−0.007	0.946	**N422(D)**	−0.697	0.835	**A443(D)**	−0.325	0.947	**D454(N)**	−0.094	0.906	**R455(D)**	−0.171	0.934	**D421(A)**	−0.115	0.909	**I389(V)**	−0.45	0.855
**S380(D)**	0.084	0.944	**N422(E)**	−0.739	0.832	**A443(E)**	−0.385	0.948	**D454(Q)**	−0.125	0.909	**R455(E)**	−0.305	0.931	**D421(A)**	−0.461	0.882	**I389(T)**	−0.522	0.846
**S380(E)**	0.065	0.937	**N422(Q)**	−0.441	0.872	**A443(Q)**	−0.278	0.925	**D454(H)**	0.016	0.894	**R455(Q)**	−0.439	0.895	**D421(A)**	−0.128	0.913	**I389(S)**	−0.378	0.84
**S380(Q)**	−0.075	0.915	**N422(H)**	−0.359	0.898	**A443(H)**	0.127	0.896	**D454(I)**	−0.287	0.886	**R455(H)**	−0.517	0.864	**D421(A)**	0.023	0.901	**I389(R)**	−0.059	0.881
**S380(H)**	−0.102	0.878	**N422(I)**	−0.484	0.912	**A443(I)**	0.056	0.905	**D454(L)**	−0.352	0.867	**R455(I)**	−0.433	0.903	**D421(A)**	−0.24	0.913	**I389(Q)**	−0.644	0.858
**S380(M)**	−0.227	0.896	**N422(L)**	−0.412	0.902	**A443(L)**	0.041	0.902	**D454(K)**	0.411	0.79	**R455(L)**	−0.401	0.92	**D421(A)**	−0.255	0.908	**I389(N)**	−0.556	0.84
**S380(F)**	−0.346	0.898	**N422(K)**	−0.067	0.909	**A443(K)**	0.028	0.848	**D454(M)**	-0.335	0.876	**R455(K)**	−0.534	0.881	**D421(A)**	0.503	0.795	**I389(M)**	−0.589	0.871
**S380(W)**	−0.466	0.86	**N422(M)**	−0.484	0.895	**A443(M)**	−0.133	0.922	**D454(F)**	−0.347	0.888	**R455(M)**	−0.473	0.885	**D421(A)**	−0.256	0.894	**I389(L)**	−0.449	0.874
**S380(T)**	−0.002	0.944	**N422(F)**	−0.512	0.879	**A443(F)**	−0.052	0.914	**D454(S)**	0.072	0.93	**R455(F)**	−0.502	0.889	**D421(A)**	−0.351	0.891	**I389(K)**	−0.054	0.893
**S380(Y)**	−0.392	0.872	**N422(S)**	−0.536	0.899	**A443(S)**	−0.246	0.931	**D454(T)**	−0.116	0.924	**R455(S)**	−0.155	0.917	**D421(A)**	0.033	0.941	**I389(H)**	−0.434	0.875
**S380(V)**	−0.292	0.905	**N422(T)**	−0.608	0.902	**A443(T)**	0.039	0.909	**D454(W)**	−0.317	0.873	**R455(T)**	−0.3	0.925	**D421(A)**	−0.428	0.872	**I389(F)**	−0.704	0.859
**S380(K)**	0.278	0.807	**N422(W)**	−0.764	0.842	**A443(W)**	−0.006	0.901	**D454(Y)**	−0.352	0.888	**R455(W)**	−0.568	0.879	**D421(A)**	−0.021	0.955	**I389(E)**	-0.793	0.83
**S380(L)**	−0.285	0.907	**N422(Y)**	−0.65	0.866	**A443(Y)**	−0.001	0.905	**D454(V)**	−0.215	0.921	**R455(Y)**	−0.586	0.879	**D421(A)**	−0.401	0.876	**I389(D)**	−0.827	0.834
**S380(I)**	−0.209	0.904	**N422(V)**	−0.479	0.899	**A443(V)**	0.054	0.909	**D454(E)**	−0.357	0.891	**R455(V)**	−0.328	0.928	**D421(A)**	−0.163	0.919	**I389(A)**	−0.294	0.854

ΔΔG*_pred_*, total predicted change in stability (kcal/mol). ΔΔG*_pred_* < 0.0 indicates a stabilizing mutation. C*_pred_*, confidence estimation, given as a value between 0.0 (not reliable) and 1.0 (highly reliable).

**Table 2:** Comparison of the binding free energy (±SEM) of mutants obtained by the MM-PBSA/GBSA method.

Mutants	MM-PBSA (kcal/mol)	MM-GBSA (kcal/mol)
A443D	−91.51 ± 0.42	−59.86 ± 0.29
S380L	−94.90 ± 0.34	−56.74 ± 0.27
A443N	−80.29 ± 0.34	−51.59 ± 0.26
N422A	−66.44 ± 0.39	−38.87 ± 0.27
N422I	−64.34 ± 0.34	−37.49 ± 0.27
N422T	−86.74 ± 0.33	−52.86 ± 0.25
S380I	−71.96 ± 0.41	−40.69 ± 0.31
D421I	−62.95 ± 0.39	−45.20 ± 0.29
I389D	−70.14 ± 0.31	−47.45 ± 0.23
I389W	−71.06 ± 0.34	−45.55 ± 0.22
I389T	−57.75 ± 0.39	−42.92 ± 0.26
D454W	−62.27 ± 0.37	−44.12 ± 0.26
D421W	−42.71 ± 0.37	−41.08 ± 0.26
**E40/ERK2**	**−75.64 ± 0.27**	**−49.50 ± 0.2**

**Table 3:** Comparison of the energetics in kcal/mol (±SEM) of mutants with E40/ERK2 obtained by the MM-PBSA/GBSA method. VDWAALS, van der Waals energy; EEL, MM electrostatic energy. The polar and non-polar contributions are EGB (or EPB) and ESURF (or ENPOLAR), respectively, for MM-GBSA (or MM-PBSA).

Mutants		A443D	A443N	S380L	S380I	N422A	N422I	N422T
**Energetics**	**VDWAALS**	−97.75 ± 0.23	−88.88 ± 0.22	−91.76 ± 0.25	−97.73 ± 0.22	−94.84 ± 0.28	−86.68 ± 0.26	−91.07 ± 0.24
	**EEL**	−626.46 ± 2.63	−589.4 ± 2.97	−673.80 ± 1.87	−521.631 ± 2.08	−441.77 ± 1.77	−457.6 9 ± 1.88	−407.50 ± 2.08
	**EGB**	672.13 ± 2.41	634.15 ± 2.86	712.20 ± 1.78	582.05 ± 1.95	503.72 ± 1.76	512.63 ± 1.82	448.52 ± 1.97
	**ESURF**	−7.78 ± 0.03	−7.43 ± 3.01	−3.38 ± 0.03	−3.38 ± 0.04	−5.97 ± 0.03	−5.75 ± 0.04	−2.81 ± 0.03
	**∆G_gas_**	−724.22 ± 2.59	−678.31 ± 3.01	−765.56 ± 1.89	−619.36 ± 2.07	−536.62 ± 1.84	−544.37 ± 1.92	-498.58 ± 2.07
	**∆G_solvation_**	664.35 ± 2.41	626.72 ± 2.86	708.82 ± 1.77	578.67 ± 1.94	497.75 ± 1.75	506.88 ±1.80	445.71 ± 1.97
	**∆G TOTAL**	−59.8 6 ± 0.29	−51.59 ± 0.26	−56.74 ± 0.27	−40.69 ± 0.31	−38.87 ± 0.27	−37.49 ± 0.27	−52.86 ± 0.25

**Mutants**		**D421I**	**I389D**	**I389W**	**I389T**	**D454W**	**D421W**	
**Energetics**	**VDWAALS**	−102.03 ± 0.2	−92.03 ± 0.22	−93.21 ± 0.22	−84.65 ± 0.22	−104.74 ± 0.27	−104.74 ± 0.27	
	**EEL**	−357.28 ± 2.08	−409.02 ± 2.03	−501.96 ± 1.85	−341.38 ± 2.02	−307.17 ± 2.28	−274.95 ± 1.61	
	**EGB**	423.76 ± 1.81	463.06 ± 1.95	558.67 ± 1.79	390.69 ± 1.95	379.18 ± 2.29	348.28 ± 1.54	
	**ESURF**	−9.65 ± 0.01	−9.04 ± 0.01	−9.04 ± 0.01	−8.77 ± 0.01	−10.18 ± 0.01	−10.06 ± 0.01	
	**∆G_gas_**	−459.31 ± 1.96	−501.06 ± 2.01	−595.18 ± 1.86	−426.04 ± 1.97	−411.92 ± 2.40	−379.30 ± 1.62	
	**∆G_solvation_**	414.11 ± 1.81	453.61 ± 1.95	549.62 ± 1.79	381.91 ± 1.95	368.99 ± 2.28	338.21 ± 1.53	
	**∆G TOTAL**	−45.20 ± 0.29	−47.45 ± 0.23	−45.55 ± 0.22	−44.12 ± 0.26	−42.92 ± 0.2	−41.08 ± 0.26	

**Table 4:** Distance, occupancy, and angle of the hydrogen bonds formed from ERK2-A443D/D421W/S380L.

System	Acceptor	Donor	Occupancy (%)	Distance	Angle
**A443D/ERK2**	ASP475-OD2	TYR222-OH	99.0	2.66	166.77
	ASP442-OD2	HIE221-NE2	84.6	2.80	154.14
	ASP454-OD1	ARG180-NH1	52.5	2.78	159.34
	VAL177-O	ARG455-NH1	50.7	2.81	154.93
	ASP224-OD2	TYR444-OH	43.9	2.70	161.74
	ASN488-O	TYR176-OH	42.7	2.76	160.74
	ASP409-OD2	LYS220-NZ	22.2	2.79	152.82
	TYR176-O	ARG455-NH	16.2	2.86	160.46
	ASN190-OD1	LYS479-NZ	3.6	2.85	154.21
**D421W/ERK2**	ASP475-OD2	TYR222-OH	99.0	2.66	166.77
	ASP442-OD2	HIE221-NE2	84.6	2.81	154.13
	VAL177-O	ARG455-NH1	50.7	2.81	154.92
	ASP454-OD2	ARG180-NH1	46.7	2.80	159.39
	ASP224-OD1	TYR444-OH	43.9	2.71	161.74
	ASN488-O	TYR176-OH	42.7	2.75	163.75
	ASP454-OD1	ARG180-NH1	38.3	2.80	157.34
	ASP409-OD2	LYS220-NZ	22.2	2.79	152.82
	TYR176-O	ARG455-NH	16.2	2.85	160.46
**S380L/ERK2**	ASP475-OD2	TYR222-OH	98.9	2.65	167.16
	ASP442-OD2	HIE221-NE2	84.4	2.80	153.80
	ASP421-OD1	ARG180-NH2	72.6	2.80	154.62
	ASN488-O	TYR176-OH	68.7	2.75	162.41
	ASP454-OD1	ARG180-NH1	55.1	2.77	160.96
	ASP224-OD1	TYR444-OH	52.8	2.70	161.61
	ASP421-OD1	ARG180NH1	49.6	2.84	149.43
	TYR176-O	ARG455-NH1	45.3	2.85	159.24
	ASP409-OD2	LYS220-NZ	14.0	2.79	154.31

**Table 5:** Type of receptor-ligand interactions in mutant complexes.

System	Acceptor	Donor	Interaction
**A443D/ERK2**	ASP475-OD2	TYR222-OH	Cation-Π
	ASP442-OD2	HIE221-NE2	Salt Bridge
	ASP454-OD1	ARG180-NH1	Salt Bridge
	VAL177-O	ARG455-NH1	Salt Bridge
	ASP224-OD2	TYR444-OH	Cation-Π
	ASN488-O	TYR176-OH	Amino-Π
	ASP421-OD1	ARG455-NH	Salt Bridge
	ASP409-OD2	LYS220-NZ	Salt Bridge
	TYR176-O	ARG455-NH	Cation-Π
	ASN190-OD1	LYS479-NZ	Salt Bridge
**D421W/ERK2**	ASP475-OD2	TYR222-OH	Cation-Π
	ASP442-OD2	HIE221-NE2	Salt Bridge
	VAL177-O	ARG455-NH1	Salt Bridge
	ASP454-OD2	ARG180-NH1	Salt Bridge
	ASP224-OD1	TYR444-OH	Cation-Π
	ASN488-O	TYR176-OH	Amino-Π
	ASP454-OD1	ARG180-NH1	Salt Bridge
	ASP409-OD2	LYS220-NZ	Salt Bridge
	TYR176-O	ARG455-NH	Cation-Π
	TRP421-O	ARG455-NE	Cation-Π
**S380L/ERK2**	ASP475-OD2	TYR222-OH	Cation-Π
	ASP442-OD2	HIE221-NE2	Salt Bridge
	ASP421-OD1	ARG180-NH2	Salt Bridge
	ASN488-O	TYR176-OH	Amino-Π
	ASP454-OD1	ARG180-NH1	Salt Bridge
	ASP224-OD1	TYR444-OH	Cation-Π
	ASP421-OD1	ARG180NH1	Salt Bridge
	TYR176-O	ARG455-NH1	Cation-Π
	ASP409-OD2	LYS220-NZ2	Salt Bridge

## Data Availability

The data presented in this study are available within the article or supplementary materials.

## Supplementary Materials

The following are available online: Figure S1: Comparison of the RMSD values of mutants and E40/ERK2; Figure S2: Comparison of secondary structures of DARPin E40 and mutants (A443D, S380L, and D421W) obtained by using (A) PredictProtein and (B) SCRATCH protein predictor web servers. (For PredictProtein, helixes are shown in blue and others in yellow; for the SCRATCH protein predictor, H is for helix and C is for others.); Table S1: Comparison of predicted ∆∆G (kcal/mol) mutants of 3ZU7 from different web-based algorithms.

## Sample Availability

Samples of the compounds are available upon request.
