# Supplementary material for: Molecular Dynamics Simulations in Designing DARPins as Phosphorylation-Specific Protein Binders of ERK2
Source: Molecules. 2021 Jul 27;26(15):4540. doi: 10.3390/molecules26154540 (PMC8347146; doi:10.3390/molecules26154540)
Supplement: Supplementary file 1 [file molecules-26-04540-s001.zip › Table S1.pdf]

Table S1. Comparison predicted ΔΔG (kcal/mol) mutants of 3ZU7 from different web- based algorithms.

ΔΔG Prediction kcal /mol)	MAESTRO	Dynamut	mCSM	SDM	DUET
**A443D**	-0.325	-0.154	-0.551	0.100	-0.255
**S380L**	-0.285	2.495	-0.463	1.430	0.114
**A443N**	-0.232	0.169	-0.215	0.090	-0.048
**N422A**	-0.517	0.005	-0.501	1.800	0.301
**N422T**	-0.608	0.400	-0.078	-0.650	0.185
**N422I**	-0.484	-0.151	-0.020	0.970	0.557
**S380I**	-0.209	2.300	-0.463	1.700	0.176
**D421I**	-0.240	1.173	0.369	0.480	0.804
**I389D**	-0.827	-0.036	0.088	-0.790	0.395
**I389W**	-0.672	-0.117	-0.592	0.150	-0.468
**I389T**	-0.522	-0.106	-0.541	-1.270	-0.383
**D454W**	-0.317	0.136	-0.102	-0.090	-0.230
**D421W**	-0.428	1.879	0.198	-0.840	-0.151

Note: Mutation prediction from servers MAESTRO, Dynamut, mCSM (mutation Cutoff Scanning Matrix), SDM (Site Directed Mutator) and DUET. *For MAESTRO, ΔΔG < 0 indicates stabilizing mutations while for Dynamut, SDM, mCSM and DUET servers, ΔΔG > 0 indicates stabilizing mutations.
