# Supplementary figures and images for: Molecular Dynamics Simulations in Designing DARPins as Phosphorylation-Specific Protein Binders of ERK2
Source: Molecules. 2021 Jul 27;26(15):4540. doi: 10.3390/molecules26154540 (PMC8347146; doi:10.3390/molecules26154540)

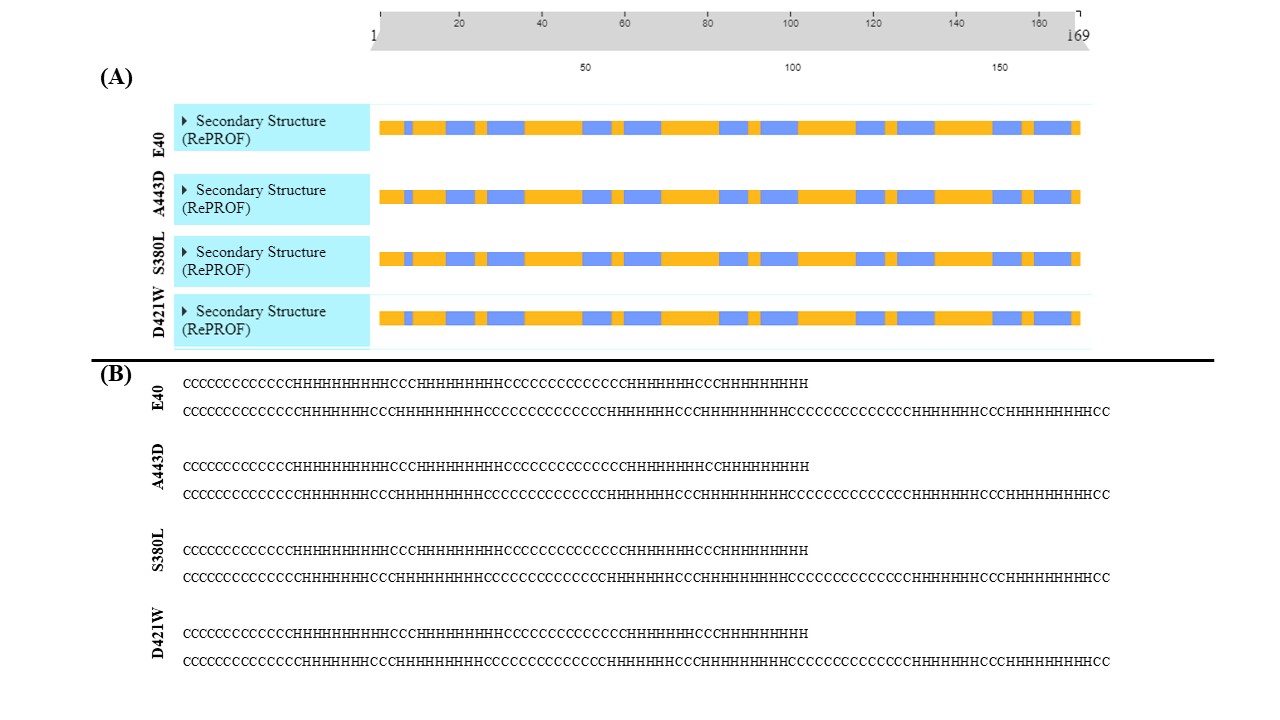

Supplement: Supplementary file 1 [file molecules-26-04540-s001.zip › Figure S2.jpg]
